# Book and Pamphlet Notices

**Published:** 1859-04

**Authors:** 


### BOOK AND PAMPHLET NOTICES.

*Cours de Physiologie Compares. Leçons professee au Musee d'Histoire Naturelle. Par M. Flourens. Paris*, 1856.

COURSE OF COMPARATIVE PHYSIOLOGY. Given at the Museum of Natural History, (Garden of Plants.) By M. FLOURENS. Paris, 1856.

When M. Flourens writes a book, science is advanced and the world benefited. This is well known to men of science, particularly to students of physiology and of natural history, and all his works might be cited as proofs. We limit ourselves to the citation of his works on the nervous system, and on the growth of bone, as sufficient for our present purpose.

The practical merit of Flourens' books consists in this, that while they are unsurpassed in accuracy and originality as works of science, they often solve and put at rest incidental questions of a popular nature, the proper understanding of which is of importance to society. Thus, his work on the brain was the basis of his *Examen de la Phrenologie*, in which the theory of bumps and big heads received its final quietus.

The present work treats of the origin and formation of living beings, of spontaneous generation, of the origin, extinction and transformation of the species of animals. Incidentally, it demolishes that fabric of speculation and fanciful views developed with so much clearness in a popular form, in the romance called the "Vestiges of Creation," a work founded on the theory of cell formation of Schwann.

It is on this account that we take this occasion to give our readers some knowledge of this work of the illustrious secretary of the Academy of Sciences. We wish, too, that our readers should perceive that the science of physiology is concerned in the solution of all these great questions which have arisen during this century; that it is not, as is sometimes asserted, the science of organs and functions only.

It is often said, that the dead are many times more numerous than the living. This is true of the species as of the individual. The extinct species far outnumber the living. Seven species of fossil hippotomi are found in the chalk beds under Paris: there is but one living species known. "There are 40,000 species of fossil shells now extinct." Is the earth, then, being depopulated? Not at all. "The number of species is daily diminishing." "The quantity of life on the globe is always the same."

What is the characteristic of each species of animal? It is the power of perpetual reproduction. A cross between the horse and ass, between a dog and a jackal, may sometimes reproduce, but never beyond the fourth degree. It is the character of hybrids or mongrels to become extinct at the fourth generation at the farthest. There is no evidence that any species of animal has ever changed its essential characteristics of form, habits or character. While the individuals change, and the elements of each are always changing, the form is perpetual. M. Flourens asserts, and no one is more competent to decide, that from the earliest historical period to the present, there is no evidence that a single species of animal has ever changed its essential characteristics.

Such being the facts, we are prepared to do justice to the theories of creation invented by ingenious speculators. A Frenchman, named Maillet, consul at Cairo in 1748, led off in the explanation of the origin of animals on the earth. Finding that appearances indicated that the earth had at an early period been covered with water, he concluded that all animals had at first been fishes. The waters retiring, left the fishes in the mud, and they crawled, hence reptiles; the flying fish became birds, etc. M. Maillet asserted that man himself was originally a fish.

All this is sufficiently ridiculous, but it is precisely the theory of Lamarck, which in the more scientific names has many supporters. Lamarck makes every thing from the monad. The monad developed becomes the polypus, and this in turn, according to the circumstances in which it is placed, originates the different species of animals. The monad of Lamarck is the cell of Schwann, which, under different circumstances, is developed into the various forms of living beings which people the earth.

In answer to this theory, M. Flourens points to the fact, that neither climate, temperature, food, mingling of blood, convulsions of nature, nor any other cause, has ever been able to change the characteristics of the species. It may become extinct from these extraneous causes, but while it lives, it retains the same form.

Hence all men are of the same species, and have a common origin. This is the conclusion to which the facts adduced incidentally led. M. Flourens, in attempting to demonstrate the unity of the human race from physiological laws, arrives at the conclusions which have formed the basis of popular belief from time immemorial.

He will, no doubt, be received as a welcome recruit in the ranks of the defenders of this doctrine; for, truth to say, it has been attacked of late years, with a degree of earnestness and vigor which have seriously shaken the faith of many in its correctness. Are the races of men, dogs, horses, and oxen, respectively of the same parentage? The question cannot be regarded as settled. As yet the weight of scientific authority seems to be in favor of the affirmative.

But, how was animal life first introduced upon the earth? Was it by spontaneous generation, or some other unexplained means?

All antiquity believed in spontaneous generation. For them everything came out of the earth. The renewed force and activity of both vegetable and animal life exhibited in the spring seem to the careless observer to prove it. Epicurus said, the earth, in its first energy, produced all animals and men. Plutarch acknowledged that in his time this energy was greatly diminished, and only produced rats. Burdach, one of the ablest of recent physiologists, believed in spontaneous generation. M. Flourens does not believe in it. He combats this doctrine with an array of facts and force of logic, which scarcely fail to carry conviction to the mind. The scientific world at the present time is almost unanimous against it. "Of all errors," says Fleurens, "this is the most absurd." "It is also the hardest to kill." It is in truth hard to kill. There are some who believe, that all the life on the earth resulted from the action of the rays of the sun upon it. Every year somebody succeeds in creating some little animals, as he thinks; and scarcely is the source of deception detected, than others, by different processes, are produced. The last instance of this kind was that of M. Pouchet, who, on the 20th Dec. last, presented a paper to the Academy of Sciences, in which he says:

"After having repeated all the serious experiments heretofore made on this subject, I came at length to those of Messrs. Schultze and Schwann." "At the present time I can certify that, by following exactly the processes pursued by them, and even by varying them, and giving them a much higher degree of precision, I have constantly obtained a positive result." "We see produced animalculæ and cryptogamia in a glass bottle, in which all trace of organic germs have first been destroyed, and where the air could only come, after having been amply washed in concentrated sulphuric acid, or after having traversed a labyrinth of fragments of porcelain at a red heat." M. Pouchet also produced his animalculæ in artificial air. We have not seen the report on this paper by the committee charged to examine it, of which M. Flourens was one. M. Pouchet is an eminent physician, and incapable of deceiving, unless deceived himself. But he will hardly gain for his views the assent of the "Imperial Academy."

Was animal life introduced upon the earth by successive creations, or all at one time?

The doctrine of successive creations is so fixed in the minds of the whole world, that it might seem absurd to call it in question. Nevertheless, M. Flourens does so; combats it, if not with success, at least with a power sufficient to cast a doubt upon it. He says, "the first traces of life are found in the transition series of formations; they appear as mollusca, crustacea, and even fishes. As we ascend in the sedimentary deposits, we meet reptiles, birds, mammifera, and even quadrimana." "The species are not presented, mixed together—each layer presents a distinct population." Hence it has been inferred that the epochs of creation corresponded to that of the successive layers.

This view supposes that present observations are perfect, that the entire earth has been explored. Nothing is further from the truth. Almost every succeeding year reveals fossils of vertebrata in layers, in which they had before been supposed not to be found. A single specimen found in the lower strata would destroy the whole theory, which, although it now holds possession of the ground, does so by a *precarious tenure*, and is liable at any time to be dispossessed by the production of a better title.

As the views of M. Flourens are new, and opposed to those generally held, we deem it proper to present an outline of his arguments in support of them. They are:

1st. The unity of the animal kingdom. There is not a double animal kingdom, one fossil and the other living. Each taken separately is but an incomplete part, reunited they make a whole, and adopt themselves each to the other, like the parts of a bas relief when restored. Thus the *plesiosaurus* is placed between reptiles and amphibia, the *pterodactyle* binds together birds and reptiles, etc. Yet the former are found as fossils, the latter living.

2d. Most naturalists, following Cuvier, in admitting successive creations, found their theory upon negative facts, as, for example, this: in the layers where reptiles are buried, no mammifera are found. Facts have already shaken, if not demolished this theory. Bones of the *mammifera*, the *quadrimana*, and even of man, *perhaps*, have been found in a fossil state, some of them in the lower strata. We say, perhaps, for M. Flourens does not assert in so positive a manner as M. de Blainville has done, that human fossil bones have been found.

3d. The resemblance, if not identity, of living and fossil species, fossil bones of elephants, horses, bears, etc., can scarcely be distinguished from those of living species. Why should we suppose them created at different periods.

M. Flourens promises to develop the proofs of his theory in detail, at some future time. At present he simply indicates them, concluding his course of lectures with the following words:

"In the times in which we live, the master no longer imposes his doctrines on his pupils; he gives them up for examination, for discussion. Meditate upon the views which I have presented; whether you accept them or not, I shall be happy to have provoked useful and conclusive researches on your part." "It is thus that each of us will have furnished a stone for that beautiful temple of science which is being erected by the nineteenth century."

---

On Poisons, in relation to Medical Jurisprudence and Medicine. By Alfred Swaine Taylor, M.D., F.R.S., Fellow of the Royal College of Physicians, etc. Second American from the second and revised London edition. Philadelphia: Blanchard & Lea. 1859. Pages 755.

Ten years ago Mr. Taylor's treatise on poisons was noticed in this Journal by Prof. Blaney. In the present edition the author professes to have brought the subject forward according to the advancement of science. Instead of giving a catalogue of poisons with their actions, he has "devoted more space to the consideration of substances which, from the frequency of their employment for murder and suicide, are of great practical importance."

This remodeling of the subject is required by the changes of the last few years.

For instance, the alkaloid strychnia was discovered forty years ago, and in eight or ten years after entered into medicinal use. Its deadly properties as a poison were known to the medical faculty, but it is only within a short time that the public have been apprized of these facts. In England, strychnia had never been used as a poison (except, *perhaps*, in one case) until the use of it by William Palmer; since then, its use for suicide and poison has been often repeated. But when the medical profession have been so educated as to distinguish the symptoms accompanying the administration of it (as a choking sensation, twitchings, and tetanic convulsions), the villain will fear to use this means of killing his victim.

The forty-three chapters of the present volume treat of the nature of poisons—their mode of action—absorption—influence of habit—classification, etc.—evidence of poisoning in the *living*

body—diseases resembling poisoning—evidence of poisoning in the *dead* body—evidence from chemical analysis—experiments on animals, etc. The author treats extensively of irritant poisons as mineral acids—oxalic acid—alkalies and alkaline salts—phosphorus—arsenic—mercury—lead—copper, etc.; and of neurotic poisons, divided into the great classes of cerebral, spinal, and cerebro-spinal poisons. Of these, the most common are opium, prussic acid, alcohol, chloroform, tobacco, strychnia, hemlock, belladonna, digitalis, and lobelia.

On page 696, in speaking of G. W. Greene, of Chicago, the author advances the opinion that there was a "failure of the medical evidence to show that death was caused by strychnia," and that the chemical analysis merely proved the existence of a medicinal dose of strychnia. But all who are cognizant of the facts will feel that no injustice was done to Mr. Greene by the verdict rendered.

The position of Mr. Taylor, as Professor of Medical Jurisprudence and Chemistry in Guy's Hospital, would be sufficient guarantee of close study and careful enunciation of the truth on this difficult subject. Besides, he is already favorably known to the American public as author of a treatise on medical jurisprudence and a former edition of this same book, which has risen to the position of a standard work. He has attained a great reputation, and occupies a niche in equal rank with Orfila and Christison. To any one who wishes to understand the subject of poisons, and especially in their relations to medicine and law, this treatise will afford great assistance. D.

